# Multifunctional human monoclonal antibody combination mediates protection against Rift Valley fever virus at low doses

**DOI:** 10.1038/s41467-023-41171-3

**Published:** 2023-09-13

**Authors:** Nathaniel S. Chapman, Ruben J. G. Hulswit, Jonna L. B. Westover, Robert Stass, Guido C. Paesen, Elad Binshtein, Joseph X. Reidy, Taylor B. Engdahl, Laura S. Handal, Alejandra Flores, Brian B. Gowen, Thomas A. Bowden, James E. Crowe

**Affiliations:** 1 Department of Pathology, Microbiology, and Immunology, Vanderbilt University Medical Center, Nashville, TN 37232 USA; 2 The Vanderbilt Vaccine Center, Vanderbilt University Medical Center, Nashville, TN 37232 USA; 3 Division of Structural Biology, Wellcome Centre for Human Genetics, University of Oxford, Roosevelt Drive, Oxford, OX3 7BN UK; 4 Department of Animal, Dairy and Veterinary Sciences, Utah State University, Logan, UT 84322 USA; 5 Department of Pediatrics, Vanderbilt University Medical Center, Nashville, TN 37232 USA

**Keywords:** Viral infection, X-ray crystallography, Antiviral agents

## Abstract

The zoonotic Rift Valley fever virus (RVFV) can cause severe disease in humans and has pandemic potential, yet no approved vaccine or therapy exists. Here we describe a dual-mechanism human monoclonal antibody (mAb) combination against RVFV that is effective at minimal doses in a lethal mouse model of infection. We structurally analyze and characterize the binding mode of a prototypical potent Gn domain-A-binding antibody that blocks attachment and of an antibody that inhibits infection by abrogating the fusion process as previously determined. Surprisingly, the Gn domain-A antibody does not directly block RVFV Gn interaction with the host receptor low density lipoprotein receptor-related protein 1 (LRP1) as determined by a competitive assay. This study identifies a rationally designed combination of human mAbs deserving of future investigation for use in humans against RVFV infection. Using a two-pronged mechanistic approach, we demonstrate the potent efficacy of a rationally designed combination mAb therapeutic.

## Abstract (web summary)

There are currently no treatment options for infection with Rift Valley fever virus (RVFV). Here, Chapman et al., show that a combination of human monoclonal antibodies targeting two different steps in early virus cell entry enhances protection in a mouse model and is effective at low doses.

## Introduction

Emerging viral outbreaks have the potential to disrupt society and impact human health.

RVFV, first identified in 1931^1^, is an emerging mosquito-borne phlebovirus in the *Phenuiviridae* family with the potential to cause widespread outbreaks in diverse geographical regions with global host and vector presence^2^. Pandemic concerns exist due to the globalization of livestock trade and the presence of large and concentrated groups of virus-naive animal hosts, such as cattle and sheep. Furthermore, mosquitoes implicated in the spread of RVFV have been found in North America and Europe^1^. These concerns are enhanced with altering global weather patterns that may enable RVFV-carrying mosquito populations to spread to new geographic regions^3–5^. Typically, RVFV outbreaks are characterized by mass livestock die-off events in which RVFV zoonotic transmission to humans occurs by infected mosquito exposures or direct contact with infected animals or animal products^6^. RVFV typically causes mild disease in humans with flu-like symptoms consisting of fever, muscle pain, joint pain, and headache. In severe cases, however, patients can present with ocular disease, meningoencephalitis, or potentially lethal hemorrhagic fever^6^. RVFV infection during pregnancy may lead to miscarriage through direct placental infection^7,8^. The World Health Organization (WHO) and the U.S. National Institutes of Health have acknowledged the high threat of RVFV to livestock and human health to prioritize research to develop vaccines and therapeutic countermeasures^9–11^. Despite the threat posed to human health and the potential of RVFV to cause a pandemic, either naturally or through a bioterrorist event^12^, no RVFV-specific vaccines or therapies have been approved.

RVFV has an RNA genome consisting of three segments: the large (L), medium (M), and small (S) gene segments^13^. The M gene segment encodes the glycoprotein precursor (GPC) and the nonstructural protein NSm which is post-translationally cleaved into the nonstructural protein NSm, Gc and Gn. These latter two glycoproteins together form an icosahedral lattice that mediates viral attachment, entry, and fusion^14–16^. The Gc glycoprotein is a class II membrane fusion protein and is shielded by the membrane distal N-terminal head region of the Gn termed Gn^H^, which consists of three domains: domain A, B, and a β-ribbon domain^17–19^. Animal vaccine trials indicate that virus neutralization mediated by antibody recognition of the Gc and Gn proteins contributes to protection from experimental RVFV challenge^20–26^. Furthermore, a limited number of human monoclonal antibodies (mAbs) have been isolated that bind to Gc and/or Gn, neutralize the virus, and provide potent pre-exposure protection and strong post-exposure therapeutic benefit in animal models of infection^27,28^.

RVFV infects a wide range of hosts. A broad range of permissive cell and tissue types has been described for RVFV^8,29–33^. Primary infection is thought to begin with the uptake of RVFV by dendritic cells, mediated by *N*-glycan recognition on Gc and Gn by the C-type lectin receptor dendritic cell-specific intercellular adhesion molecule-3-grabbing non-integrin (DC-SIGN)^34,35^. Heparan sulfate also has been shown to facilitate RVFV infection^36^. Recently, low-density lipoprotein receptor-related protein 1 (mouse Lrp1/human LRP1), a receptor that helps facilitate protein uptake, was described as a host entry factor to support RVFV infection^37–40^. LRP1 is highly conserved between humans and livestock [60–98% at the amino acid level], albeit with limited conservation to mosquitoes (30–45%)^41^] and is expressed at varying levels in diverse cells and tissues^42^. Following attachment, RVFV enters host cells via caveola-mediated endocytosis^43^. The conservation and broad expression of LRP1 may explain in part the diversity of hosts and infected tissues after exposure to RVFV.

Human Gn-specific antibodies block the binding of the Gn tetramer to Huh7 cells and block virus attachment to Vero cells, but the mechanism of blockade is not fully defined^27^. For other viruses, the mechanism of blocking virion attachment to cells has been inferred by measuring competition of antibodies and receptor protein for binding to a viral attachment factor or a receptor binding domain from the attachment protein^44–47^. We considered possible mechanisms for this activity: (1) direct blocking of Gn binding to the LRP1 receptor by competition for binding to the same site, (2) indirect blocking by steric hindrance for an antibody binding to a nearby but distinct site, (3) indirect blocking by bivalent binding of both arms of an IgG causing reduced access to the receptor binding domain, or (4) indirect blocking by antibody-induced conformational changes causing an allosteric change that alters the conformation of Gn. Here, we sought to gain an understanding of how potent human antibodies function to block the initial step of infection to characterize mechanisms of action and inform therapeutic approaches to RVFV infection.

Fusion inhibition is a common mechanism by which potently neutralizing antibodies against class II fusion proteins inhibit viruses, and we previously showed that mAb RVFV-140 inhibits RVFV in this manner^28,48–50^. The endosomal membrane fusion event that is a critical step in the virus entry pathway is triggered by exposure of the fusion protein to low pH, which alters the viral surface protein. For RVFV, this change allows the Gc fusion loop to extend and interact directly with the host membrane^51,52^.

RNA viruses can mutate against selective pressure from mAb monotherapy due to high polymerase error rates and rapid escape variant selection^53^. A combination mAb therapeutic approach that interrupts multiple points of entry early in the RVFV viral life cycle may be of additional value and mediate a superior beneficial effect than combinations blocking a single virus entry step. We studied two potent antibody classes with distinct functional profiles, with one class blocking attachment of Gn domain A to LRP1 using a bivalent mode of IgG binding and the second class inhibiting membrane fusion. We then combined representative mAbs from the two distinct classes of antibodies to create a combination entry blockade therapeutic for RVFV that works through two distinct mechanisms. Low doses of this antibody combination, which blocks both attachment and fusion, protected against a lethal challenge of RVFV in a murine infection model.

## Results

### Human Gn-specific neutralizing antibodies do not directly block RVFV Gn interaction with LRP1 receptor protein

**Fig. 1:**
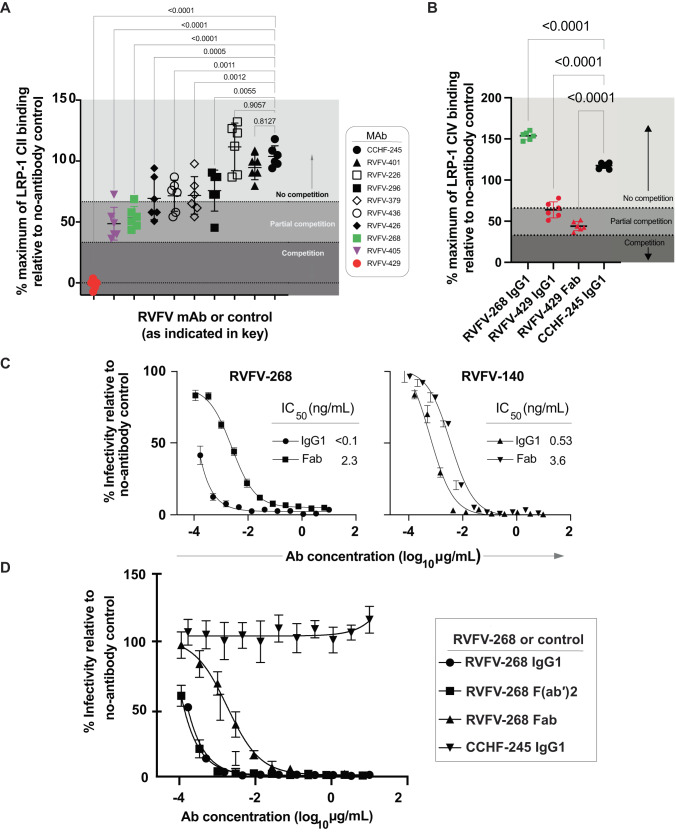
RVFV-specific Gn mAbs do not compete directly with LRP1 Clusters. **A**, **B** Using BLI, we loaded his-tagged Gn onto HIS1K biosensors and then associated human antibodies or RVFV-429 Fab. Following this, we then bound **A** LRP1 Cluster II or **B** LRP1 Cluster IV. The values shown are a percentage of maximal signal from LRP1 Cluster II or IV binding relative to the no-antibody control. A similarly prepared human mAb to another bunyavirus designated CCHF-245 was used as the negative control mAb. The values are indicated by the gray scale. Competition was defined as less than 33% residual binding (dark gray), partial competition was greater than 33% but less than 66% residual binding (medium gray), and non-competitive percentages were greater than 66% residual binding of receptor fragments relative to the no-antibody control (light gray). Values shown are representative of three technical replicates from two independent experiments. RVFV-401, RVFV-296, RVFV-379, RVFV-436, RVFV-426, RVFV-268, RVFV-405 bind Gn domain A, RVFV-226 binds Gn domain B, and RVFV-429 binds an epitope away from the other antibodies where K197 was identified as a critical residue. Critical residues to map these antibodies were previously described^28^. Data are presented as mean values +/− SD and statistical test was done by using an ordinary one-way ANOVA test corrected for multiple comparisons. **C** Neutralization of RVFV strain MP-12 by IgG1 or Fab forms of RVFV-268 or RVFV-140. Serial dilutions of IgG1 or Fab molecules were mixed with 100 infectious units of MP-12 and allowed to incubate on Vero cells for 3 days. The Fab molecule starting dilution was 2/3 of that of IgG1 to normalize the molarity of binding sites. IC_50_ values were calculated using a three-parameter nonlinear fit. Curves reflect data from duplicate wells in three independent experiments. Data are presented as mean values +/− SEM. **D** RVFV-268 F(ab′)2 retains potent activity like IgG1. Similarly, Fab and F(ab′)2 dilutions began at 2/3 of the IgG1 starting concentrations. IC_50_ values were calculated using a three-parameter nonlinear fit. Curves reflect data from duplicate wells in three independent experiments. Data are presented as mean values +/− SD. Source data are provided as a Source data file.

We first sought to further define how Gn domain-A-specific antibodies function to neutralize RVFV and block attachment to host cells given the discovery of a RVFV receptor. Here we used biolayer interferometry (BLI) to measure the ability of previously isolated and described human antibodies targeting Gn to block the interaction between LRP1 and recombinant Gn (note, that recombinant Gn is out of context from the native virion and the Gc–Gn heterodimer interaction). First, using a recombinant LRP1 Cluster II protein, we observed a low level of blocking between Gn and LRP1 Cluster II in the presence of neutralizing human antibodies. A non-neutralizing antibody (RVFV-429, Supplementary Fig. S1) completely blocked the interaction between LRP1 Cluster II and Gn (Fig. 1A). The blocking effects of recombinantly expressed RVFV-429 IgG1, RVFV-429 Fab and RVFV-268 IgG1 were measured on the same platform with LRP1 Cluster IV, a repeat region of LRP1 that dominates the interaction between Gn and LRP1. The potent Gn domain A antibody, RVFV-268, did not block the interaction of LRP1 Cluster IV and Gn. In contrast, RVFV-429 partially blocked the interaction between Gn and LRP1 Cluster IV when tested either as a Fab or as a full-length IgG1 molecule (Fig. 1B). These data suggest that potent Gn domain A antibodies (such as RVFV-268, RVFV-379, and RVFV-426) block the attachment of virus to cells without directly competing for the binding of Gn to the LRP1 receptor. Thus, failure to fully block the LRP1-Gn interaction is not a requirement for potent neutralizing activity. It is likely that some antibodies may be discovered in future that fully block interaction with LRP-1, although we have yet to identify one.

### Potent Gn domain A-specific antibodies require bivalent binding to RVFV strain MP-12 for potent neutralizing activity

We next sought to determine if the activity of potent antibodies that represented the two functional classes of antibodies, such as RVFV-268 (Gn domain A class) and RVFV-140 (fusion inhibitory class), was mediated by the bivalent nature of full-length IgG1 or by steric hindrance. We first measured the affinity of the Gn-targeting mAbs from our previously reported antibody panel with RVFV-140 (recognizing an unknown epitope) and RVFV-326 (Gc-targeting) as the negative controls. We observed that most of the antibodies in the panel, from weakly neutralizing antibodies to potent antibodies, are bound with high affinity. However, RVFV-142 exhibited potent neutralizing activity despite binding with low affinity. Taken together, these findings suggest that the values for binding affinity in the assays we developed and those from neutralization assays do not correlate. Instead, epitope recognition dominates the observed neutralization (Supplementary Fig. S2).

Next, we produced Fab forms of RVFV-268 and RVFV-140 and compared the neutralization potency curves for IgG1 versus Fab molecules. We observed a stark reduction in the half maximal inhibitory concentration (IC_50_) value of the Fab of RVFV-268 compared to that of the IgG1 molecule. In contrast, the fusion-inhibiting antibody RVFV-140 did not exhibit as large a reduction in activity (Fig. 1C). Next, we created F(ab′)_2_ forms of the antibodies (by cleaving IgG with IdeS enzymes to remove the Fc domain). The F(ab′)_2_ form of RVFV-268 retained the potent activity of the full-length IgG1 molecule (Fig. 1D). Although the Fab form of RVFV-268 still neutralized the live attenuated RVFV MP-12 vaccine strain, the loss of activity observed indicates that bivalent binding [mediated by IgG or F(ab′)_2_], rather than steric interference of the Fc domain (possible only with an IgG), drives the potent activity of RVFV-268.

### RVFV-268 and non-neutralizing rRVFV-429 recognize spatially distinct epitopes on RVFV Gn

**Fig. 2:**
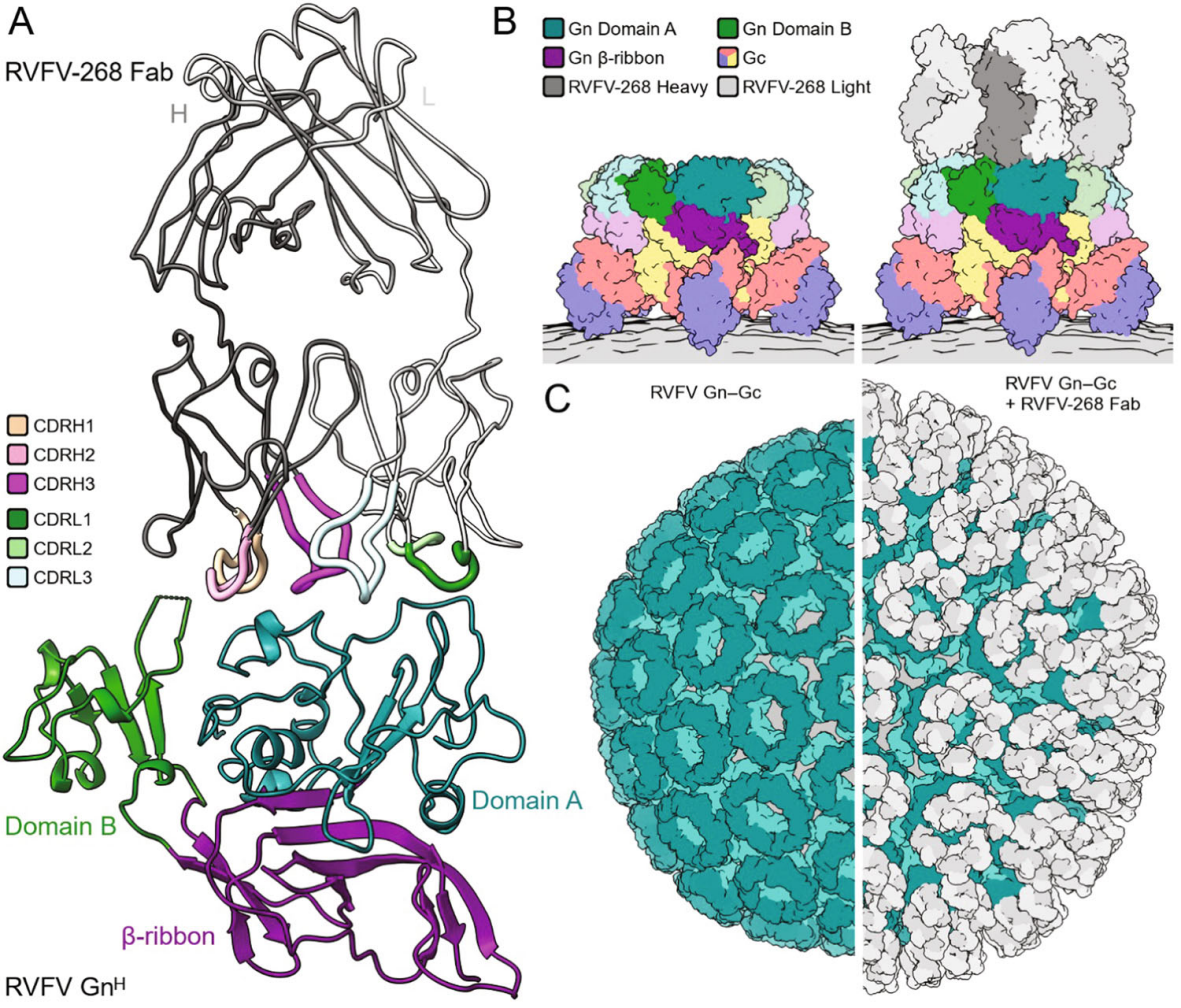
Structure of the N-terminal ectodomain region of RVFV Gn^H^ in complex with the Fab of RVFV-268. **A** Crystal structure of the Fab region of mAb RVFV-268 bound to the N-terminal ectodomain head region of RVFV Gn (Gn^H^). Proteins are colored as defined in the key. **B** A model of Fab RVFV-268 bound to the higher-order pentameric RVFV Gn−Gc assembly (PDB ID 6F9F). Proteins are colored as defined in the key. **C** (left) The icosahedral assembly of RVFV Gn−Gc (surface representation, PDB ID 6F9B). RVFV Gn is colored teal and RVFV Gc is colored light teal. (right) A model of Fab RVFV-268 (white surface) bound to the icosahedral RVFV Gn−Gc assembly (surface representation). The virion membrane is colored gray.

We next sought to visualize the binding mode of RVFV-268 and RVFV-429. The Fab region of RVFV-268 was crystallized in complex with RVFV Gn^H^, (residues 154−469) and the structure was solved to 3.5-Å resolution (Fig. 2A and Supplementary Table 1). One complex of RVFV Gn was observed in the asymmetric unit of the unit cell. Consistent with previous epitope mapping studies^28^, the RVFV-268 epitope maps to amino acid residues located in domain A of RVFV Gn^H^. The RVFV-268 epitope occludes ~650 Å^2^ of buried surface area and overlaps with previously structurally characterized human mAbs, namely, mAb R12, R13 and R15^27^ (Supplementary Fig. S3). Interestingly, RVFV-268 exhibits a relatively large degree of somatic mutations from its putative germline precursor (Supplementary Fig. S4), relative to mAbs R12, R13, and R15. This observation suggests that this region of RVFV Gn^H^ is a commonly targeted site of vulnerability on the RVFV surface at both early and later stages of the adaptive immune response to RVFV infection. The structure of RVFV-268 in complex with Gn^H^ reveals how antibodies encoded by the V3 lambda gene can acquire affinity to the Gn^H^ surface. Both previous studies identified potent antibodies that are encoded by variable gene segments of the V3 lambda gene family^27,28^.

We then utilized low-resolution negative stain electron microscopy (nsEM) to understand the region of RVFV Gn^H^ recognized by RVFV-429. Binary and trinary complexes consisting of RVFV Gn^H^−RVFV-268 and RVFV Gn^H^−RVFV-268−RVFV-429 were formed and imaged, respectively, and single-particle reconstructions were derived to ~19-Å and ~22- Å resolution, respectively (Supplementary Fig. S5). Although low resolution, these combined reconstructions are consistent with non-competition binding data^28^ and reveal that mAbs RVFV-268 and RVFV-429 recognize non-overlapping epitopes on RVFV Gn^H^.

To identify the mode of RVFV-268 recognition in the context of the higher-order assembly of RVFV Gn−Gc glycoproteins, we overlayed the RVFV Gn^H^−RVFV-268 structure onto a cryoEM-derived Gn−Gc assembly model (Fig. 2B, C). This analysis reveals that the RVFV-268 epitope is accessible in the context of the icosahedral assembly of RVFV, where no conformational changes to Gn−Gc ultrastructure are required to facilitate binding and there is space for each RVFV Gn to be targeted simultaneously (Fig. 2C). Additionally, consistent with the importance of bivalency in potent neutralization (Fig. 1), we note that RVFV-268 epitopes are in close proximity, such that both Fab regions of the mAb can interact simultaneously with RVFV Gn. Finally, given that our nsEM-derived indicates that binding of RVFV-268 and RVFV-429 occurs at non-overlapping sites of RVFV Gn^H^ (Supplementary Fig. S5), it seems plausible that the RVFV-429 epitope locates to a site on RVFV that is not accessible on the mature virion surface and that conformational changes to the Gn−Gc lattice are required for mAb recognition. Further work should be done to fully interrogate the accessibility of this epitope.

### Neutralization is mediated by antibodies from two distinct functional classes against RVFV MP-12 vaccine strain on diverse human cell lines

**Fig. 3:**
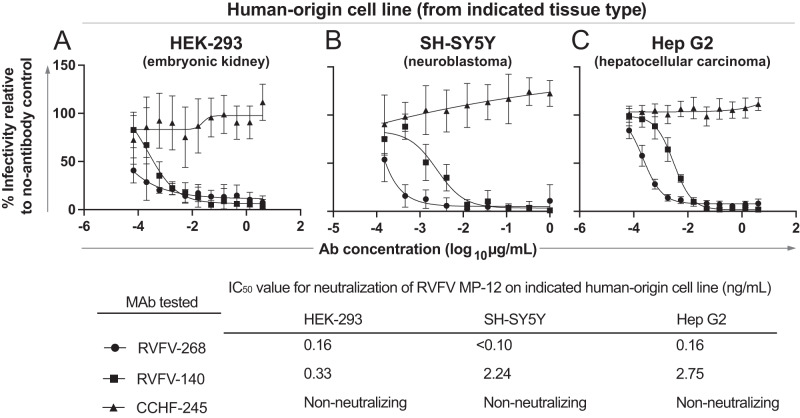
RVFV-268 and RVFV-140 retain potent neutralizing activity against RVFV strain MP-12 on a diverse set of human cell lines. The capacity of RVFV-268 and RVFV-140 to neutralize RVFV strain MP-12 was tested on **A** HEK-293 (human embryonic kidney), **B** SH-SY5Y (human neuroblastoma), or **C** HepG2 (human hepatocellular carcinoma) cell lines. Antibody solutions were serially diluted and mixed with 100 infectious units of RVFV strain MP-12 for 1 h at 37 °C. The mixture was added to cells and allowed to incubate for 72 h. Cells were fixed and stained. Foci were counted using a C.T.L. spot counter instrument. The experiment was performed in technical triplicate with three independent experiments. Values are expressed as a percentage of foci in no-antibody controls and mAb CCHF-245 was used as the isotype-matched negative control antibody. The MP-12 neutralization assay was performed twice with three technical replicates in each assay. Results were similar between biological replicates; data shown are the IC_50_ values of combined data from two independent experiments. IC_50_ values were calculated using a three-parameter nonlinear fit. Data are presented as mean values +/− SD. Source data are provided as a Source data file.

To confirm the activity of potent Gn domain A and fusion-inhibiting antibodies against MP-12 on diverse human cell lines, we tested RVFV-268 and RVFV-140 against the RVFV MP-12 strain on HEK-293 (kidney), SH-SY5Y (neuroblastoma), or HepG2 (liver) cell culture monolayers. These cells are permissive to RVFV infection and represent a few of the major target tissue types affected during natural infection. Both RVFV-268 and RVFV-140 showed potent neutralizing activity against RVFV vaccine virus strain MP-12 on each of the cell lines tested (Fig. 3A–C), indicating that both mechanisms of neutralization described above function in diverse cell lines and suggesting that the route of viral entry may be similar across tissues. RVFV-268, the attachment-blocking antibody, left a small residual fraction of non-neutralized virus in the HEK-293 and HepG2 cell line experiments. In contrast, the fusion-inhibiting mAb RVFV-140 neutralized 100% of viral infection across all cell lines tested.

### Mutations that cause loss of binding of RVFV Gn-specific antibodies

**Fig. 4:**
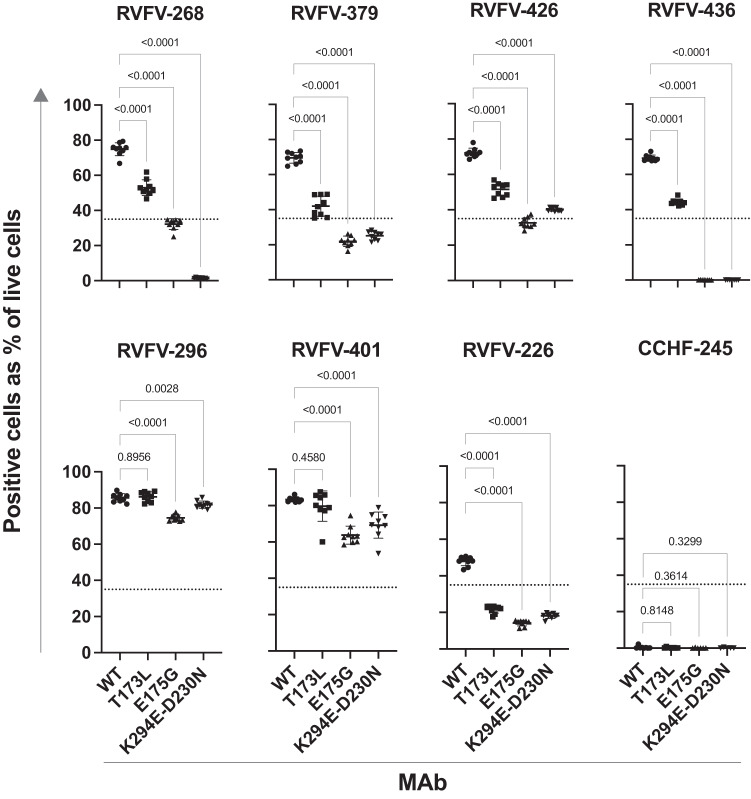
Antibodies are sensitive to naturally occurring mutations. The binding of these Gn-specific antibodies to Gn proteins with previously identified naturally occurring mutations^27^ was tested next. Gene constructs encoding Gn with WT or variant sequence were synthesized and transiently expressed in suspension cells. Cells were stained for viability and fixed and then subsequently permeabilized for staining with an individual antibody and then with anti-human Fc PE-conjugated antibodies. The variants tested were: T173L, E175G, and K294E-D230N. Cells were assessed on the high-throughput IntelliCyt iQue flow cytometric screener. Cells were gated for viability and values are expressed as a percentage of positive cells (signal over 10^5 on the BL2-H channel) over the total count of viable cells. The data shown represents technical triplicate values over three independent experiments. RVFV-296 was not sensitive to any mutation, revealing that the antigens were expressed at similar levels. CCHF-245 was used as a negative control. Data are presented as mean values +/− SD and statistical test was done by using an ordinary one-way ANOVA test corrected for multiple comparisons. Source data are provided as a Source data file.

A major concern with using mAb monotherapy to prevent or treat RNA virus infections is the possibility of selecting antibody escape mutant variant viruses. Certain antibodies may be difficult to escape if critical contact residues for binding are important for viral fitness. Here, we attempted to isolate a variant of MP-12 virus after a single round of replication that was not neutralized by either RVFV-140, RVFV-268, or the combination of the two mAbs in vitro. We did not observe escape for any of these treatments, suggesting that all infectious viruses in the input inoculum were fully neutralized by each mAbs or the combination (Supplementary Fig. S6). We then used the Gn sequences of previously identified RVFV field strains with naturally occurring variant residues in Gn^27^ to assess if the activity of these antibodies is sensitive to mutations in those virus variants. RVFV-268 lost partial ability to bind to Gn proteins with T173L or E175G mutations and lost all capacity to bind to a Gn with K294E-D230N mutations. RVFV-379 and RVFV-426, two antibodies that are less potently neutralizing but that compete for binding to a similar binding site as RVFV-268, similarly lost some binding to T173L or E175G mutants while retaining a reduced level of binding to K294E-D230N (Fig. 4 and Supplementary Fig. 7). We were unable to test the fusion-inhibiting antibody RVFV-140’s sensitivity to these mutations on the Gn surface since this antibody does not bind to monomeric Gn proteins.

### Low-dose monotherapy in vivo using potently neutralizing antibodies from two distinct classes of mAbs

**Fig. 5:**
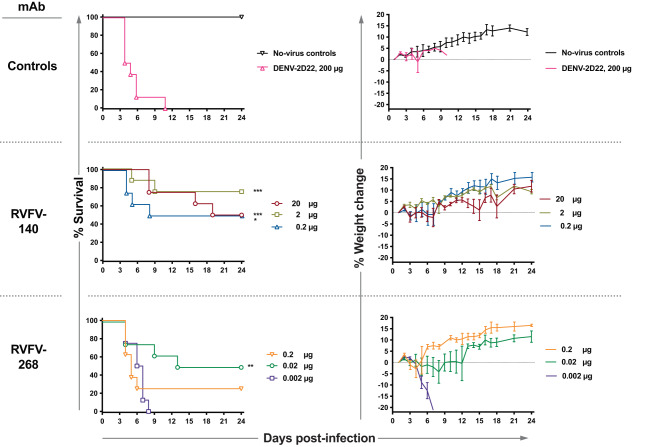
Monotherapy is effective at low doses against RVFV infection in the BALB/c mouse model. A single dose of mAb was administered by the IP route to mice (*n* = 8 per group) at 2 d.p.i. The virus was administered by SC inoculation of 300 PFU of RVFV strain ZH501. RVFV-268, RVFV-140, or DENV-2D22 (an isotype-matched negative control mAb) were tested in a sequential dose-down study design in this stringent therapeutic model of infection. Doses administered for RVFV-140: 20 µg, 2 µg, or 0.2 µg. Doses administered for RVFV-268: 0.2 µg, 0.02 µg, or 0.002 µg. Kaplan–Meier survival plots were statistically analyzed using a log-rank (Mantel–Cox) test where mAb-treated animals (***P < 0.001, **P < 0.01, *P < 0.05) were compared to animals treated with the DENV-2D22 negative control mAb. Weight graphs reflect group means standard error of the means of the percent change in weight of animals relative to body weight obtained the day after virus challenge. Sham-infected no virus controls are shown. Median survival for each condition tested: no-virus control—Undefined; DENV-2D22—4.5 days; 20 µg RVFV-140—21.5 days; 2 µg RVFV-140—Undefined days; 0.2 µg RVFV-140—16 days; 0.2 µg RVFV-268—5 days; 0.02 µg RVFV-268—18.5 days; 0.002 µg RVFV-268—6.5 days. P values for each condition tested compared to the DENV-2D22 control treated group using a Log-rank (Mantel-Cox) test: no-virus control—0.0018; 20 µg RVFV-140—0.0002; 2 µg RVFV-140—0.0009; 0.2 µg RVFV-140—0.0345; 0.2 µg RVFV-268—0.3770; 0.02 µg RVFV-268—0.0084; 0.002 µg RVFV-268 –0.4403. Source data are provided as a Source data file.

We previously reported the therapeutic efficacy of the antibodies RVFV-268 or RVFV-140 in the BALB/c mouse model of infection with RVFV wild-type (WT) strain ZH501 at 2 days post-infection (d.p.i). Here, we extended those studies to determine the lowest dose of antibody that is effective in therapeutic models in vivo. We used a mixed-sex murine model of infection that was established previously^28^, measuring animal survival and viral titers in liver, spleen, and blood following virus challenge and antibody treatment. Subcutaneous administration of infectious RVFV was followed by the administration of a mAb on 2 d.p.i. by the intraperitoneal (IP) route with a series of increasingly lower doses of RVFV-140 or RVFV-268. We tested 200 µg (10 mg/kg) of each antibody and dosed down to 60 or 20 µg for RVFV-140 and 20, 2, or 0.2 µg for RVFV-268 (Supplementary Fig. S8A). In all cases, we observed statistically significant protection. A minor amount of variability was observed (the highest doses did not always protect 100%). At most, 2 out of a group of 8 animals died. We recovered virus from one of the moribund mice and sequenced the portion of the genome of the virus encoding Gn. Some single nucleotide polymorphisms were observed in the viral RNA from the brain and liver that differed from the input virus, although the changes were not uniform (Supplementary Fig. S8B). It is possible that the late-stage death we observed is due to virus replication in the central nervous system causing neurological disease, since we recovered replicating virus from this mouse’s brain. This phenomenon has been previously observed and reported when small molecule inhibitors are used as experimental antiviral drugs^25,54^. We followed this study with treatment with even lower doses of antibodies: RVFV-140 at 20, 2 µg, or 0.2 µg; RVFV-268 at 0.2, 0.02, or 0.002 µg. RVFV-140 provided significant protection at each of the three doses tested, and RVFV-268 provided significant levels of protection at a 0.02 µg dose (Fig. 5). The patterns of reduction of viral titers observed reflected the general trend of protection, with the higher doses typically causing reduced viremia (Supplementary Fig. S9). The relative protection conferred by the antibodies corresponded to the in vitro IC_50_ values for neutralization in that the more potently neutralizing the antibody, the lower the dose needed to achieve statistically significant protection in vivo. The Gn domain A attachment-blocking antibody RVFV-268 has a lower IC_50_ value for neutralization and protected animals in vivo at a lower dose than the less potently neutralizing fusion-inhibiting mAb RVFV-140.

### Combination therapy with two mAbs using complementary mechanisms of action

**Fig. 6:**
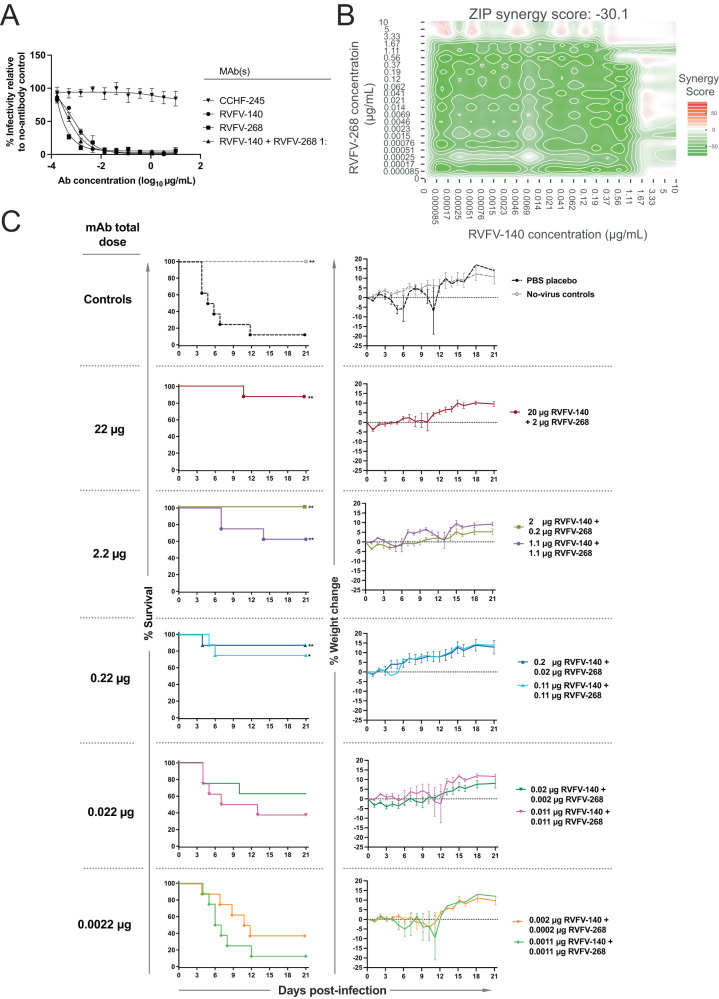
A non-synergistic pair of antibodies is effective therapeutically at low doses. **A** RVFV-140 and RVFV-268 do not synergize for neutralization. Neutralization curves for RVFV strain MP-12 were developed using the standard neutralization assay. RVFV-140, RVFV-268, or the combination of the two antibodies at a 1:1 ratio were tested in serial dilutions against 100 infectious units of RVFV strain MP-12. Data are presented as mean values +/− SEM. **B** Combinations of RVFV-268 and RVFV-140 are not synergistic. Calculated synergy scores are shown. Serial dilutions of each antibody were mixed and incubated with RVFV vaccine strain MP-12 and subsequently added to a Vero cell monolayer. Neutralization was calculated relative to no-antibody control wells and values were imported to SynergyFinder using a zero interactions potency (ZIP) statistical model. Delta scores >5 indicate synergy and values <-5 indicate antagonism. Two independent experiments were performed, and the data are averages of values across experiments. **C** Combination therapy in stringent mouse model of RVFV infection protects at low mAb doses. Combinations of mAbs RVFV-140 and RVFV-268 were administered once by the IP route to mice (*n* = 8 per group) at 2 d.p.i. Infection occurred by SC inoculation of 100 PFU of RVFV strain ZH501. Combinations of RVFV-140 / RVFV-268 and PBS vehicle were tested in sequential dose down in this stringent therapeutic model of infection. Doses administered were a tenfold sequential dose down of either a 1:1 or a 1:10 ratio of RVFV-140 and RVFV-268, respectively. Kaplan–Meier survival plots were statistically analyzed using a log-rank (Mantel–Cox) test where treated animals (**P < 0.01, *P < 0.05) were compared to animals treated with PBS negative control. Weight graphs reflect group means and standard error of the means of the percent change in weight of animals relative to the weight the day of virus challenge. Sham-infected no virus controls are shown. *P* values for each condition tested compared to the PBS control treated group using a Log-rank (Mantel-Cox) test: no-virus control—0.0096; 20 µg RVFV-140 + 2 µg RVFV-268—0.0014; 2 µg RVFV-140 + 0.2 µg RVFV-268—0.0096; 0.2 µg RVFV-140 + 0.02 µg RVFV-268—0.0041; 0.02 µg RVFV-140 + 0.002 µg RVFV-268—0.0507; 0.002 µg RVFV-140 + 0.0002 µg RVFV-268—0.1183; 1.1 µg RVFV-140 + 1.1 µg RVFV-268—0.0095; 0.11 µg RVFV-140 + 0.11 µg RVFV-268—0.0133; 0.011 µg RVFV-140 + 0.011 µg RVFV-268—0.2285; 0.0011 µg RVFV-140 + 0.0011 µg RVFV-268—0.6706. Source data are provided as a Source data file.

A combination of mAbs for RNA virus infection is typically preferable to monotherapy, given the capacity of these viruses to escape neutralization. To this end, we combined RVFV-268 and RVFV-140 to incorporate both attachment blockade and fusion-inhibiting activities into a candidate therapeutic mixture. Some combinations of mAbs to a viral single protein exhibit synergistic neutralization^48,55,56^, but we did not detect synergy in vitro for the mAbs RVFV-268 and RVFV-140 (Fig. 6A, B). We then used the lowest protective doses achieved above in the monotherapy studies to design a dose-down study with the mAb combination. The study tested either a 1:1 or a 10:1 ratio combination of RVFV-140 and RVFV-268 in progressively lower total doses. Subcutaneous challenge with infectious RVFV was followed by the administration of treatment on 2 d.p.i. by the IP route with sequential dose-down of RVFV-140 + RVFV-268 in combination. The combination provided statistically significant protection at 0.22 µg total dose administered in the lethal 2 d.p.i. BALB/c mouse model (Fig. 6C). Furthermore, we observed that the 10:1 ratio (RVFV-140:RVFV-268) dosing scheme tended to be more therapeutic than the 1:1 dosing scheme. Similarly, viral titers determined for target organs appeared lower at the 10:1 ratio and were significantly reduced up to 2.2 µg total dose (Supplementary Fig. S10). At the 0.22 µg total dose, viremia was not significantly reduced, although we still observed significant protection. In these cases, it is likely the antibodies mediate protection in part by delaying the onset of high-grade viremia, allowing the animal to use its own primary immune response against the virus, thereby ensuring survival. Further studies are needed to better understand this phenomenon, but the observations already suggest that sterilizing immunity may not be required for protection in this animal model and that delaying the onset of viremia may provide therapeutic benefits. Overall, this dual mechanism combination therapeutic approach against RVFV infection was remarkably effective against lethality even at exceedingly low therapeutic doses.

## Discussion

Here we demonstrate a principle that may enhance the effectiveness of antiviral antibody combinations, which is to incorporate mAbs that possess different mechanisms of action and inhibit different aspects of the virus life cycle (attachment and fusion). A common approach in antibody drug development efforts for RNA viruses is to develop combinations that resist escape and, when possible, to achieve synergy between the functional activity of combined mAbs^55–57^. The result here was that we identified a combination of two human antibodies that protects against a fatal disease in an animal model at very low doses; the projected protective human dose for a 70 kg adult on a per kg basis would be a remarkable total dose of <1 mg. At the outset of this study, we aimed to further our knowledge of antibody mediated neutralization and protection against RVFV in order to add to the foundational knowledge of these types of antibodies that were previously described^27,28^.

Competition-binding data with protein domains of the recently identified RVFV receptor LRP1 and the negative stain EM studies suggests that an epitope other than the surface where RVFV-268 binds on Gn domain A mediates the association between the receptor and LRP1. We used a BLI assay incorporating recombinant head domain protein of Gn immobilized to a sensor, which is displayed out of context from the native virion, limiting the interpretation of the assay. However, the narrow LRP1 receptor protein may access an alternative site to that recognized by RVFV-268, possibly comprising the β-ribbon domain between two Gn protomers on the viral surface. Although Gn domain A antibodies can block attachment and exhibit potent neutralizing activity^27,28^, antibodies of this nature do not appear to directly compete with LRP1 receptor clusters for binding to monomeric Gn. This observation suggests that RVFV-268, which lost activity when the IgG form was converted to a Fab, uses a bivalent mode of binding to cross-link Gn protomers on the viral surface, concealing the access point by which the LRP1 receptor may be entering. We observed a lack of strict relationship of affinity of binding of Fabs to soluble viral protein reagents. This finding suggests that antibodies with low apparent affinity for binding to Gn protein may accomplish high avidity of binding to virion particles using both arms of the IgG molecule.

Both attachment-blocking and fusion-inhibiting classes of mAbs retained their potent neutralizing activity across diverse cell lines derived from distinct target organs, suggesting that the viral entry pathway is conserved across diverse tissues. Interestingly, we observed a small residual fraction of infection in high concentrations of the Gn domain A attachment-blocking antibody RVFV-268 in HEK-293 and HepG2 cell lines. The mouse receptor-associated protein (mRAP) is a natural ligand that binds LRP1 and competes with Gn. Previous studies have shown that mRAP can prevent infection by blocking Gn from associating with LRP1, thus producing a neutralizing effect. Furthermore, mRAP on HEK-293 and HepG2 cells also leaves a residual fraction of infection at high concentrations^37^. Conversely, RVFV-140, a fusion inhibiting antibody, did not leave a residual fraction of non-neutralized virus, despite its higher IC_50_ value for neutralization (i.e., lower potency). These observations further confirm that the two classes of antibodies we studied in detail maintain phenotypic differences in neutralization.

We chose RVFV-268 as the lead antibody in this study based on its potent activity against RVFV. However, additional similar mAbs that directly compete for binding with RVFV-268, such as RVFV-379 and RVFV-426, may display superior characteristics in terms of resistance to escape. Further studies are needed to determine whether these antibodies retain potent neutralization against mutated strains. We were unable to isolate infectious virus that escaped neutralization from RVFV-140, RVFV-268, or the combination of the two mAbs in a single-round infection assay. More stringent passaging of the virus and selection may be needed for the selection of variants with the wild-type virus compared to the use of the more genetically stable MP-12 vaccine strain^58^ to select for authentic virus escape mutants.

We first sought to determine if representative antibodies from each of the Gn domain A and fusion-inhibiting antibody classes provided statistically significant therapeutic protection in the lethal mouse model of RVFV infection at low doses. We observed that low doses of a representative mAb from each of the antibody classes protected animals at lower doses than did RVFV-140. Interestingly, we observed some viremia in animals that received low doses of antibodies but still had significant levels of protection, indicating that sterilizing immunity may not be required for therapeutic protection and delaying high titers of viremia may aid in protection. We suspect that suboptimal doses for uniform protection may allow for the virus to replicate at low levels and eventually enter the central nervous system (CNS). CNS-related RVFV disease has been observed after the initial virus has been cleared in the liver, when small molecular inhibitors were used as a treatment^25,54^. We suggest that the use of mAbs against RVFV in humans should be dosed accordingly to prevent not only viral replication, but also late-stage neurological viral replication. We plan to follow up our studies with a more rigorous assessment of CNS-related disease and viral sequences by not only quantifying serological and tissue-specific antibody levels at time of viral administration, but also assessment of viral loads and antibody levels in various tissues at the time of euthanasia.

RVFV-140 is less potent than RVFV-268 by about 10-fold in terms of the IC_50_ value for neutralization. In the animal studies, we observed a similar phenomenon, as a 10:1 ratio dosing structure provided consistently superior results to the 1:1 ratio of mAbs. This finding is consistent with the observation that RVFV-140 and RVFV-268 do not synergize for neutralization in vitro.

Here, we show the rational design of a two-mAb combination incorporating potently neutralizing RVFV antibodies that possess different modes of action and target different steps in virus entry offers a therapeutic regimen effective in vivo at doses as low as 10 µg/kg. This rational principle for antibody combination development may enable the development of an antibody-based countermeasure against Rift Valley Fever virus related infection.

## Methods

### Cell culture

Vero CCL-81 (monkey, female), cell lines were obtained from the American Type Culture Collection (ATCC CCL-81), Vero E6 (ATCC CLR-1586), and HEK-293 (ATCC CRL-3216 human, female) were maintained at 37 °C in 5% CO_2_ in Dulbecco’s Modified Eagle Medium (DMEM; Thermo Fisher Scientific) containing 10% (v/v) heat-inactivated FBS (HyClone), 10 mM HEPES pH 7.3, 1 mM sodium pyruvate, 1× non-essential amino acids, and 100 U/mL of penicillin–streptomycin. HepG2 cells were obtained from the American Type Culture Collection (ATCC; catalog number HB-8065) and maintained at 37 °C in 5% CO_2_ in EMEM with 10% (v/v) FBS. SH-SY5Y cells were obtained from the ATCC (catalog number CRL-2266) and maintained in at 37 °C in 5% CO_2_ in a 1:1 mix of EMEM:F12 medium with 10% (v/v) FBS. Expi293F cells (Thermo Fisher Scientific; catalog number A1452) were maintained at 37 °C in 8% CO_2_ in Expi293F Expression Medium (Thermo Fisher Scientific; catalog number fA1435102). Mycoplasma testing of cell cultures was performed monthly using a PCR-based mycoplasma detection kit (ATCC, 30–1012K), and all tests were negative during the time of study.

### Viruses

RVFV vaccine strain MP-12 was used at Vanderbilt University. The virus was passaged and titrated by plaque assay in Vero E6 cells. RVFV ZH501 animal studies were conducted at Utah State University in biosafety level-3 enhanced (BSL-3+) approved facilities using appropriate powered air-purifying respirators and personal protective equipment.

### Murine models of RVFV infection

Seven- to eight-week-old male and female BALB/c mice were obtained from Charles River Laboratories (Wilmington, MA). Mice were housed in microisolator cages and provided water and food ad libitum. The mouse RVFV challenge efficacy studies were approved by the Utah State University Institutional Biosafety Committee and conducted in Select Agent-approved animal (A)BSL-3+ facilities.

### Virus neutralization assay

Virus neutralization assays were performed in the foci-forming assay (FFA) format using MP-12 vaccine strain of RVFV. Plates for HEK-293 cell culture monolayers were pre-coated with poly-D-lysine overnight and washed before cells were added. Virus (100 PFU per Vero cell monolayer) was incubated with increasing concentrations of mAb in triplicate for 1 h at 37 °C, and then each suspension was added to a monolayer of either Vero, SHSY-5Y, HepG2, or HEK-293 cells in a 96-well plate for 1 h at 37 °C in the appropriate medium for each cell type. Following incubation, a 1:1 mixture of fully supplemented (5% FBS) DMEM (HEK-293 or Vero cells), EMEM (HepG2 cells), or 1:1 mix of EMEM:F12 (SH-SY5Y cells) and 2.4% methylcellulose mixture was added onto the cells. After a 3-day incubation for HepG2, Vero, or SH-SY5Y cells or a 2-day incubation for HEK-293 cells in 5% CO_2_ at 37 °C, cell monolayers were fixed for 1 h with 1% paraformaldehyde and stained with a 1:3,000 dilution of mAb-1D8 (BEI Resources) for 1 h in 2% milk in PBS-T. Following primary incubation and washing, a 1:3000 dilution of anti-mouse HRP-conjugated secondary antibody (Promega, W402B) was added for 1 h in the same buffer. Cells were washed and stained with TrueBlue^TM^ peroxidase substrate (SeraCare5510-0030) for 30 min. Cells were washed in dH_2_O, imaged on a BioSpot CTL plate reader, and foci were counted using its associated software (7.0.18.1). The percent relative infection was determined based on the virus-only control. IC_50_ values were determined using a sigmoidal, 4PL nonlinear fit analysis in Prism software version 9 (GraphPad).

To assess synergistic neutralization of RVFV-268 and RVFV-140, the antibodies were mixed at a 1:1 molar ratio before performing a neutralization assay with the MP-12 virus strain. Neutralization was determined by comparing treated wells versus no-antibody control wells. Synergy scores were calculated using the SynergyFinder software (R-3.8.2)^59^ with a zero interactions potency (ZIP) statistical model. Delta scores >5 indicate synergy and values <−5 indicate antagonism.

### Antibody production and purification

For hybridoma-derived mAb, clonal cells were grown in 75 cm^2^ flasks to 70% confluency in hybridoma growth medium (ClonaCell-HY medium E from STEMCELL Technologies, 03805). The hybridoma cells were grown to exhaustion in Hybridoma-SFM (1×) serum-free medium (Gibco Hybridoma-SFM, Invitrogen, 12045084) in four 225 cm^2^ area flasks. Exhausted hybridoma supernatant was harvested after one month. For the recombinant mAb production, the genes of heavy and light chains were synthesized into cDNA and cloned into a full-length IgG1 DNA plasmid expression vector^60^. The heavy and light chains were transformed into *Escherichia coli* cells to produce large amounts of DNA. Following the manufacturer’s protocol, plasmids encoding heavy and light antibody chains were transiently transfected into Expi293F cells to produce mAb proteins. Secreted IgGs were purified from filtered supernatants by affinity chromatography using protein G columns (GE Life Sciences, Protein G HP columns) on an ÄKTA pure instrument. Purified mAbs were processed by buffer-exchanging into PBS, filtering using sterile 0.45 μm Millipore filter devices, concentrated, and stored at −80 °C. Recombinant mAbs were used for all in vivo experiments, and designated mAbs were either hybridoma or recombinant derived in vitro experiments.

### LRP1 Cluster II and IV competition with human antibodies

For assessing the capacity of human antibodies to Gn to block the RVFV Gn-LRP1 interactions, we employed a competition-binding assay using the BLI platform on an Octet Red96 instrument (Pall FortéBio, Menlo Park). After rehydrating the HIS1K biosensors in kinetics buffer for 15 min, a 60 s baseline step was used. Following this, his-tagged Gn was loaded on the biosensor at 20 µg/mL for 1000 s. A brief 30 s baseline step was used, and following this, human mAbs or RVFV-429 Fab were allowed to associate to Gn at 40 µg/mL for 150 s. Without using another baseline step, LRP1 Cluster II (Recombinant human LRP1 cluster II Fc Chimera Protein, CF (2368-L2-050)) or LRP1 Cluster IV (Recombinant human LRP1 cluster IV Fc Chimera Protein, CF (5395-L4-050), R&D Biosystems) was allowed to associate to the Gn-antibody complex for 1500 s at 40 µg/mL. The timepoint where maximum signal was achieved for the no-antibody control was chosen post-LRP1 Cluster II/IV binding. The individual values for each antibody consider the off-rate of each antibody by subtracting the signal from the no-receptor control, which was performed with every condition respectively. Competition was defined as residual binding of the LRP1 clusters <33% of the maximal no-antibody control. Partial competition was defined as >33% and <66% residual binding of the LRP1 Clusters compared to no-antibody control. Greater than 66% residual binding of LRP1 Cluster II or IV relative to no-antibody control was defined as non-competitive.

### F(ab) fragment production

DNAs encoding heavy and light chains of antibody fragments were inserted into DNA plasmid expression vectors, processed, and transiently expressed similarly as full-length IgG expression. Fab fragments were purified using CaptureSelect CH1-XL columns (Thermo Fisher Scientific) on an ÄKTA pure instrument. Final Fab fragments were buffer-exchanged to PBS, concentrated, and frozen in an ethanol-dry ice bath and shipped to Oxford University from Vanderbilt University.

### Neutralization assays with F(ab) and F(ab′)_2_

Recombinantly produced RVFV-268 IgG1 was diluted in PBS and cleaved to F(ab′)_2_ fragments using the FabRICATOR (IdeS) FragIT SmartEnzymes kit by Genovis, per the manufacturer’s protocol. The F(ab′)_2_ fraction was taken and tested for binding to Gn recombinant using an Octet BLI assay. Virus neutralization for matching F(ab) or F(ab′)_2_ fragments was compared to the neutralizing activity of the corresponding full-length IgG1 accounting for molecular weight and valency of the molecules.

### Gn recombinant protein production

For structure determination, a construct encoding the RVFV Gn^H^ (residues 154−469; UniProt accession no. P21401) was cloned into the pHLsec expression vector^19,61^ with a C-terminal His_6_-tag. The construct was transiently transfected into human embryonic kidney (HEK) 293T cells in presence of the alpha-mannosidase inhibitor, kifunensine^62^. Supernatant was dialyzed using an ÄKTA FLUX against buffer (10 mM Tris, pH 8.0, 150 mM NaCl) following centrifugation. Protein was purified by nickel immobilized metal affinity chromatography with a 5 mL HisTrap column and isolated by size exclusion chromatography (SEC) with a Superdex 200 increase 10/30 column. For binding studies, a gene encoding the ectodomain of RVFV Gn (GenBank accession no. JQ068143.1 [https://www.uniprot.org/uniprotkb/H9BSP3/entry], residues 154–469) was synthesized, cloned, and expressed in SF9 cells (Thermo Fisher Scientific 11496015) as described previously^27,28,63^. The Gn recombinant protein was isolated by metal affinity chromatography on HisTrap Excel columns (Cytiva) as previously described^27,28,63^.

### BLI affinity measurements

For kinetic assays with BLI of Gn-specific-mAbs on an Octet Red96 instrument (Pall FortéBio, Menlo Park) was used. After hydrations of tips in kinetics buffer for 15 min, a 60 s baseline step was performed. Recombinant histidine-tagged Gn was immobilized to HIS1K biosensor tips (FortéBio) at 10 µg/mL in kinetics buffer (FortéBio) and loaded for 200 s. After a 30 s baseline step, antibody was allowed to associate to Gn for 300 s in a serial dilution scheme starting at 200 nM at a 1:2 dilution. Following association, a 1000 s dissociation step was performed in kinetics buffer. Data extrapolation of the equilibrium dissociation constant values was performed with curve-fitting on the Analysis HT 12.2.0.2 software. Reference wells for the dissociation of antigen from the biosensor tips were used to subtract background from the association and dissociation steps. For the curve fits, a global fitting using a 1:1 model with Savitzky–Golay filtering was performed.

### Crystallization and structure determination of Fab-268 alone and in complex with RVFV Gn^H^

Purified Fab-268 was concentrated to ~10 mg/mL and crystallized at room temperature using the vapor diffusion method^64^ in a precipitant containing 0.1 M citric acid pH 3.5, 20% w/v polyethylene glycol (PEG) 1500 and 4% v/v (+/−)-2-methyl-2,4-pentanediol. Purified RVFV Gn^H^ and Fab-268 were complexed on ice for 30 min in a 1.2:1 molar ratio and subsequently purified by SEC with the Superdex 200 increase 10/30 column. The purified RVFV Gn^H^- Fab-268 complex was then concentrated to ~12 mg/mL and crystallized at room temperature using the vapor diffusion method in a precipitant containing 0.1 M bis-tris pH 5.5, 25% w/v PEG 3350. Fab-268 crystals were cryoprotected in the precipitant containing 25% PEG 2000 and RVFV Gn^H^–Fab-268 crystals were cryoprotected in precipitant containing 25% glycerol prior to flash freezing in liquid nitrogen.

X-ray crystallography data were collected at Diamond Light Source on beamline i04 with a Dectris Eiger2 XE 16M detector using Diamond Light Source Generic Data Acquisition software (9.21). Diffraction data were processed with XIA2 (v3.8.0-g3d57088-dials-3.8)^65^ to 1.6 and 3.5 Å for unbound Fab-268 and RVFV Gn^H^–Fab-268, respectively. For both structures, molecular replacement was performed using PHASER^64^. To solve the structure of unbound Fab-268, the structure of a human Fab (PDB: 5ZMJ)^66^ was used as a search model. To solve the structure of the RVFV Gn^H^−Fab-268 complex, unliganded RVFV Gn^H^ (PDB: 6F8P)^19^ and the refined unbound Fab-268 were used as search models. The structures were iteratively built in COOT (v0.9.4.1)^67^ and refined in Phenix (v1.19.2_4158)^68^. MolProbity was used to assess the quality of the models^69^. Crystallographic data collection and refinement statistics for the two structures are presented in Supplementary Table 1.

### Structural representations

ChimeraX (v1.2.5)^70^ was used for preparation of figures.

### Accession codes

Atomic coordinates and structure factors of RVFV-248 Fab apo and RVFV Gn^H^ complexed with RVFV-248 Fab have been deposited in the PDB (8AWM and 8AWL). Further information and requests for resources and reagents should be directed to and will be fulfilled by the Lead Contact, James E. Crowe, Jr. (james.crowe@vumc.org). Materials described in this paper are available for distribution for nonprofit use using templated documents from Association of University Technology Managers “Toolkit MTAs,” available at: https://autm.net/surveys-and-tools/agreements/material-transferagreements/mta-toolkit.

### Negative-stain electron microscopy

Electron microscopy imaging was performed with RVFV-Gn protein in complex with Fab forms of mAbs RVFV-268 and RVFV-429. Recombinant forms of RVFV-Gn were expressed and purified as described above. Fabs forms of RVFV-268 and RVFV-429 were expressed and purified as described above or digested from IgG if needed. Complexes were generated by incubating the recombinant proteins with the two corresponding Fabs in a 1:1.2 (antigen:Fab) molar ratio. A 3 μL volume of the sample at ~10 μg/mL concentration was applied to a glow-discharged grid with continuous carbon film on 400 square mesh copper electron microscopy grids (Electron Microscopy Sciences). Grids were stained with 2% uranylformate^71^. Images were recorded on a Gatan US4000 4k × 4k CCD camera using an FEI TF20 (TFS) transmission electron microscope operated at 200 keV and control with SerialEM^72^. All images were taken at ×50,000 magnification with a pixel size of 2.18 Å/pixel in low-dose mode at a defocus of 1.5 to 1.8 mm. The total dose for the micrographs was 33 e/Å2. Image processing was performed using the cryoSPARC software package (3.3.1)^73^. Images were imported, CTF-estimated and particles were picked automatically. The particles were extracted with a box size of 200 pix and binned to 100 pix (4.36 Å/pixel) and multiple rounds of 2D class averages were performed to achieve clean datasets. The final dataset was used to generate an initial 3D volume and the volume was refined for the final map at the resolution of ~18 Å. Model docking to the EM map was done in Chimera (ChimeraX v1.3)^74^. Chimera software was used to make all figures with PDB: 5Y0W for RVFV Gn.

### Selection of antibody mutant viruses using real-time cell analysis (RTCA) of cellular impedance

Using a label-free cellular impedance method (xCELLigence; Agilent, RTCA software 2.1.0) we continuously monitored cytopathic effect indicating non-neutralized virus growth in the presence of saturating neutralizing concentrations of mAbs RVFV-140, RVFV-268, or the combination of the two mAbs^75–78^. Antibody solutions at 1 µg/mL total concentration were mixed 1:1 in 5% FBS-supplemented DMEM with RVFV strain MP-12 (~35,000 PFU per well) and incubated for 1 h at 37 °C and then added to cells. Two controls were included, a virus-only (no-mAb) and medium-only (no-virus) mixture. Selection and amplification of virus mutants that were not neutralized by the antibodies is detected when a drop in cellular impedance occurs in a well during a 96-h incubation with continuous real-time measurements. Since we did not observe any well to exhibit a drop in impedance, we were unable to identify a mutant virus using this methodology with RVFV strain MP-12 for mAb RVFV-140, RVFV-268, or the combination of the two mAbs.

### Loss of binding to recombinant Gn protein containing naturally occurring mutations

Plasmids containing cDNA inserts encoding diverse sequences of RVFV Gn were used to transiently express Gn molecules in Expi293F cells using expression method and mutations previously reported^27^. The MMKVIWFSSLICFVIQCSG peptide sequence was linked at the 5′ end of the encoded Gn protein as a signal peptide. The cDNA encoding a WT Gn protein was based on the sequence corresponding to GenBank accession number JQ068143.1 [https://www.uniprot.org/uniprotkb/H9BSP3/entry]^27^. Naturally occurring mutations (T173L, E175G, or K294E-D230N) on the Gn surface were introduced into the WT background, synthesized, and expressed transiently in Expi293F cells per the manufacturer’s protocol. Transfections were allowed to proceed for 48 h. Cells were then spun down and frozen at 10 million/mL in medium with 10% DMSO. Cells were thawed, spun down, and resuspended in PBS. Cells were stained with Live/Dead fixable violet dead cell stain (Thermo Fisher Scientific; L34955) per the manufacturer’s protocol. After staining and washing, cells were fixed using Cytofix/Cytoperm (Becton Dickinson and Co; 554714) and diluted in perm/wash buffer to 50,000 cells per 30 µL and added to a 96-well V-bottom plate. Antibodies were diluted to 20 µg/mL in perm/wash buffer and added to fixed cells for 30 min at room temperature. Cells were washed with 100 µL of perm/wash buffer, spun down, and then stained with goat anti-human IgG-PE (SouthernBiotech; 2040-09) at a 1:500 dilution in perm/wash buffer for 30 min at room temperature. Cells were then washed with 100 µL of perm/wash buffer and spun down and resuspended in 30 µL of FACS buffer. Staining was analyzed using an iQue flow cytometer (Intellicyt v9.0). Background values were determined from binding of labeled secondary mAb to untransfected Expi293F cells. Cells were gated for viability and the positive signal was gated using a similarly prepared human mAb to an unrelated antigen (clone CCHF-245) as a negative control. Results were expressed as the percent of positive cells over total viable cell counts.

### RVFV challenge in mice and viral titer

Therapeutic assessment of mAb was performed in groups of 7–8-week-old male and female mixed cohort BALB/c mice^79^ (*n* = 8 per the treatment group (4 per group for day 3 sacrifice for viral tiers) and *n* = 5 for sham-infected (2 sacrificed for viral titer controls)). Animals were inoculated with 300 PFU for individual mAb experiments and 100 PFU for combination of mAbs experiment of RVFV (ZH501 strain) by the SC (subcutaneous) route. Animals were treated once IP on 2 d.p.i. Human mAb DENV 2D22 (specific to an unrelated target, dengue virus) or PBS was used as the negative control. Mice were monitored daily from 0 to 21 d.p.i. for survival and body weight. Mice were euthanized 21 d.p.i or when moribund following IACUC-approved protocols.

Viral titers were assayed using an infectious cell culture assay, as previously described^80^. Here, a volume of tissue homogenate or serum was diluted and added to triplicate wells of Vero cell monolayer cultures. Viral cytopathic effect (CPE) was determined 7 days after plating to calculate 50% endpoints. Lower limits of detection (LOD) were 1.49 log_10_ 50% cell culture infectious dose (CCID_50_)/mL in serum and 2.1 log_10_ CCID_50_/g in tissue. In samples presenting with virus below the LOD, the representative value of LOD was assigned for analysis.

### Isolation and sequencing of virus from moribund mouse

Brain and liver tissue was extracted from mouse that had been treated therapeutically with mAb RVFV-140 and had succumbed 16 days post-infection from late-stage neurological demise. Input virus was also tested in parallel in the following steps. Tissue virus was used to inoculate confluent Vero 76 cell monolayer cultures, and RNA was isolated from the culture supernatant using Trizol LS (Invitrogen) and DNA/RNA Shield and Viral RNA buffer (Zymo Research) per the manufacturers’ specifications. Isolated RNA was then assessed for complete inactivation before further processing. RNA was suspended in water and RT-PCR amplified. The entire M segments were amplified using the following RT-PCR primers: RVFM-AFwd, 5′-ACACAAAGACGGTGC-3′, and RVFM-ARev, 5′-ACACAAAGACCGGTGC-3′^81,82^. RT-PCR products were processed on the Illumina NovaSeq6000 instrument using a paired-end 150 technique per the manufacturer’s protocol. Amplicons were shredded with a Covairs column. Data was mapped to the reference sequence (Genbank ID: DQ380200) using Geneious software (V2020.1.2).

### Quantification and statistical analysis

Kaplan–Meier survival plots were analyzed using the Mantel–Cox log-rank test. Differences between groups were analyzed by the Fisher’s exact (two-tailed) test. Viremia and mean day-to-death were compared using a one-way ANOVA with Dunnett’s posttest to correct for multiple comparisons test. Weight data are represented as the group mean and standard error of the percent change in weight of surviving animals relative to starting weight. Technical and biological replicates are indicated in the methods and figure legends. Error bars in figures represent SD. Statistical analyses were performed using Prism v9 (GraphPad RRID: SCR_002798).

### Reporting summary

Further information on research design is available in the Nature Portfolio Reporting Summary linked to this article.

### Supplementary information

Supplementary Information

Reporting Summary

### Source data

Source data

## Data Availability

Atomic coordinates and structure factors of RVFV-248 Fab apo and RVFV Gn^H^ complexed with RVFV-248 Fab have been deposited in the Protein Data Bank (PDB IDs: 8AWM and 8AWL). All relevant data for each main text figure is available within the figures or Supplemental Data. The raw data generated in this study are provided in the Source data file. Source data are provided with this paper.
