# Supplementary material for: Multifunctional human monoclonal antibody combination mediates protection against Rift Valley fever virus at low doses
Source: Nat Commun. 2023 Sep 13;14:5650. doi: 10.1038/s41467-023-41171-3 (PMC10499838; doi:10.1038/s41467-023-41171-3)
Supplement: Supplementary file 1 — Supplementary Information [file 41467_2023_41171_MOESM1_ESM.docx]

**Supplementary Figures and Tables for:**

**Multifunctional human monoclonal antibody combination mediates protection against Rift Valley fever virus at low doses**

This file contains:

Supplementary Figures 1- 10

Supplementary Table 1

**Supplementary Figure 1. Recombinant RVFV-429 does not neutralize RVFV strain MP-12.**

Recombinant RVFV-429 was prepared and diluted serially before mixing with 100 infectious units of RVFV strain MP-12. After 1 h incubation at 37°C, the mixture was added to a Vero cell monolayer and cells were allowed to incubate prior to fixation and counting. The assay was performed in biological and technical triplicates with mAb CCHF-245 included as the mAb negative control. Data were analysed using a sigmoidal, 4PL nonlinear fit analysis in Prism software version 9 (GraphPad). Data are presented as mean values +/- SEM.

**Supplementary Figure 2. RVFV-specific mAbs affinity for binding to Gn.**

We used BLI to assess affinity of binding of Gn-specific human antibodies to his-tagged Gn immobilized on HIS1K sensors. Serially-titrated antibodies (nM as indicated by color in the key) were allowed to bind to Gn, and their off-rates were assessed. Data were analyzed using curve-fitting on the Analysis HT 12.2.0.2 software, and black lines indicate the curve fit using a 1:1 model with Savitzky-Golay filtering. The calculated *K*_D_ for each antibody is shown with each chart. Data are from at least two independent experiments, but the charts shown represent results from a single run. The first dashed line represents antibody binding for 300 s starting at 290 sec. The second dashed line represents the beginning of the dissociation stage for 1,000 sec starting at 590 sec. ND indicates that binding was not detected.

**
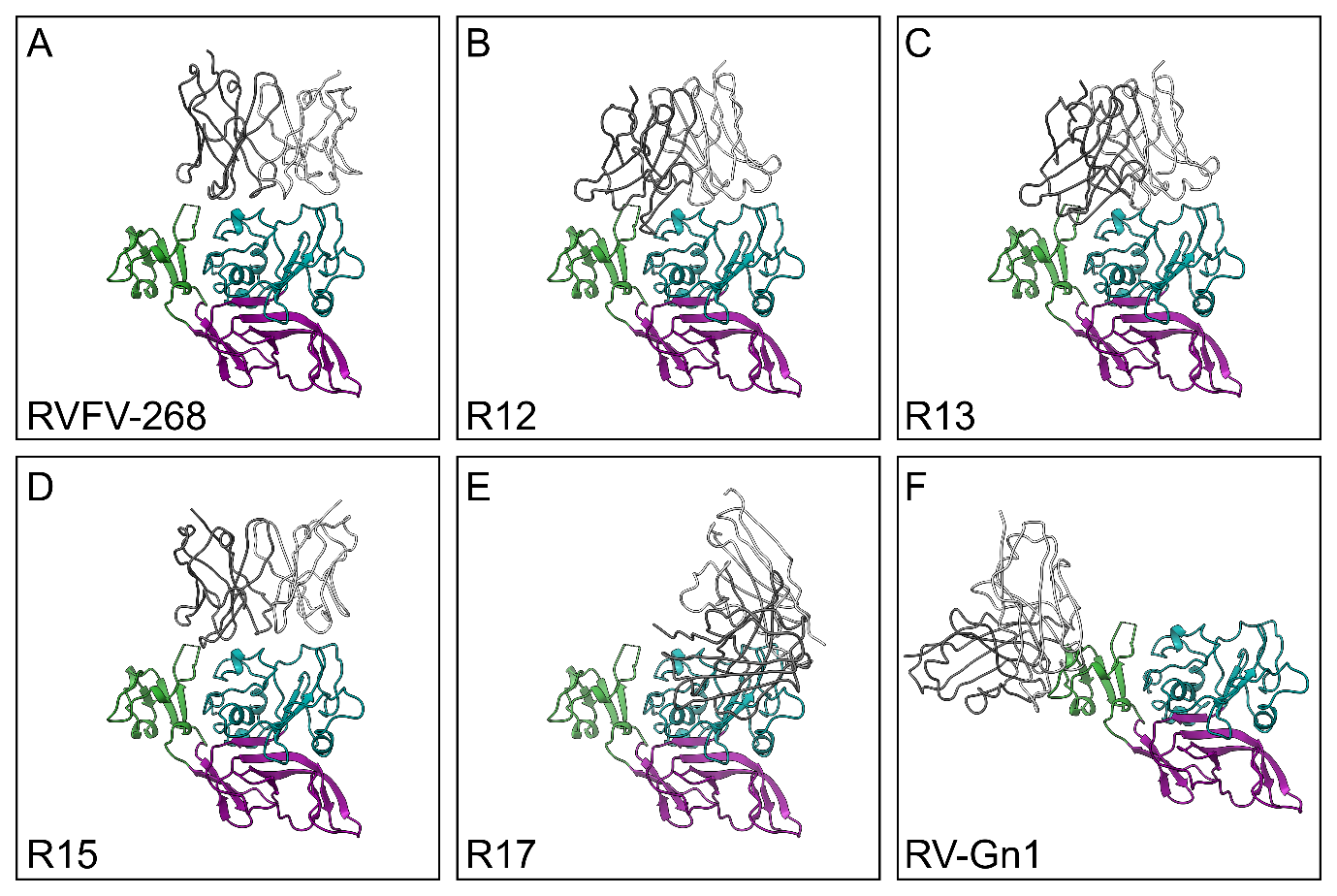
**

**Supplementary Figure S3. Structures of previously reported Fabs recognizing the RVFV Gn^H^ ectodomain.** Panel of six Fabs (A-F), including RVFV-268, in complex with the RVFV Gn^H^ ectodomain (27, 83). All panels display the antigen in the same orientation and the different domains of Gn^H^ are color coded as in the main manuscript (teal, domain A; green, domain B; purple, β-ribbon). Fabs shown in panels A through E engage Gn^H^ at domain A, where RV-Gn1 (panel F) interacts with Gn at domain B. The heavy or light chains of the Fabs are indicated in grey or white, respectively. PDB: PDB files used for comparison: PDB: 6IEK (27), PDB: 6IEA (27), PDB: 6IEB (27), PDB: 6IEC (27), and PDB: 6I9I (83).

**Supplementary Figure S4. The variable genes encoding RVFV-268 exhibit many somatic mutations from the inferred germline precursor genes.** The antibody variable gene sequences of RVFV-268 were aligned with the inferred germline gene segment sequences. Sequences are annotated based on international ImMunoGeneTics information system^®^ standards (http://www.imgt.org). FR indicates framework regions; CDR indicates complementarity-determining regions. Heavy and light chain sequences are shown separately. Periods (.) represent conserved amino acids in the sequence encoding RVFV-268 compared to the germline gene segment sequence, and dashes (-) represent gaps.

**
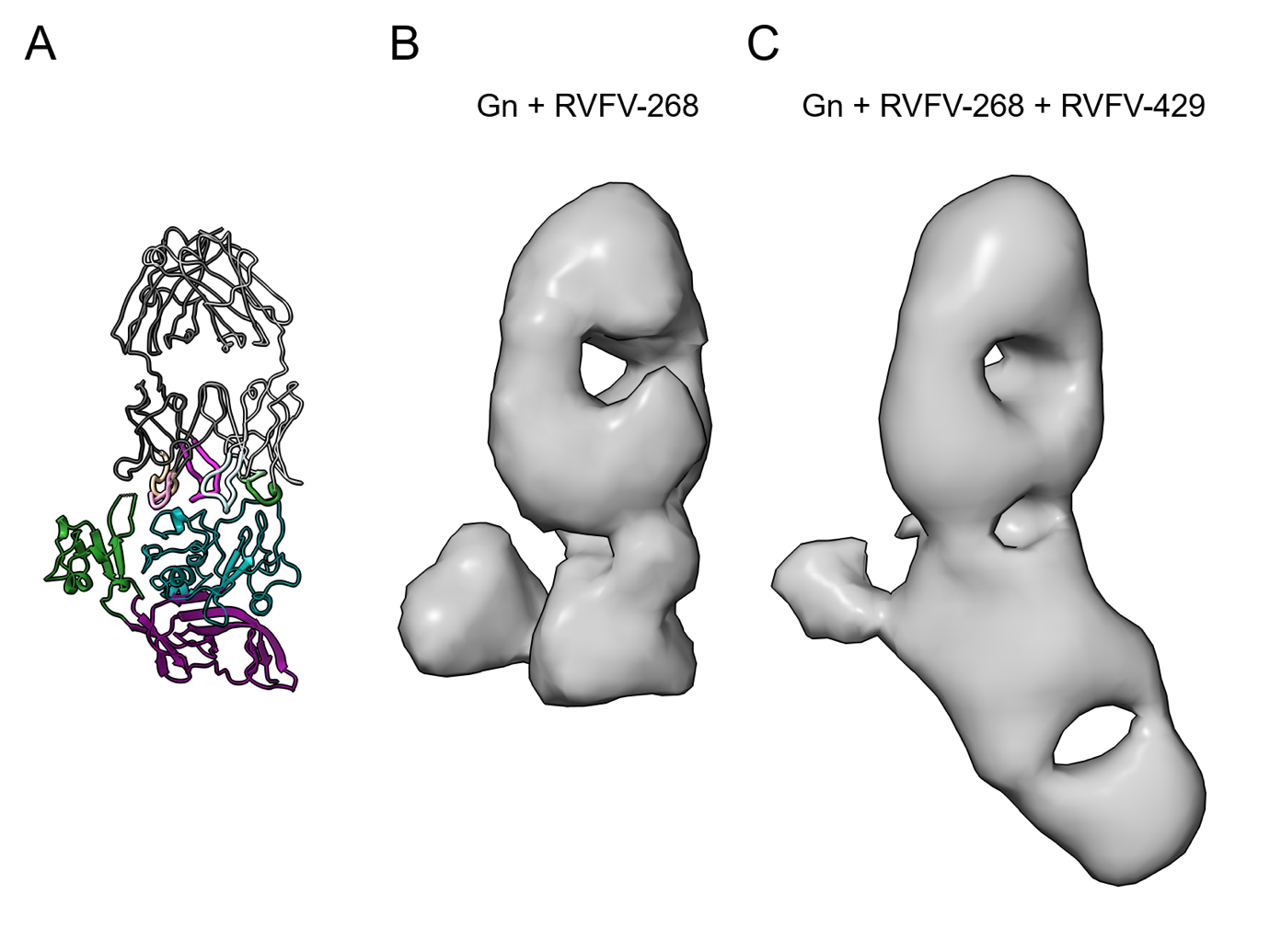
**

**Supplementary Figure S5. Low-resolution negative stain electron microscopy indicates that RVFV-268 and -429 recognize distal epitopes on RVFV Gn^H^. A)** Cartoon representation of the RVFV GnH-Fab268 crystal structure, with the different domains of Gn indicated as in Supplementary Figure S3 and CDR loops in distinctive colors. The heavy or light chain of Fab RVFV-268 are indicated in grey or white, respectively. **B**) Density model of the GnH-Fab268 complex acquired by negative stain EM. Orientation of the model is aligned with the crystal structure displayed in panel A. **C**) Density model of the Gn^H^-Fab268-Fab429 complex acquired by negative stain EM shown at the same relative orientation as panels A and B. Fab429 and Fab268 interact non-competitively with distal epitopes on the Gn^H^ antigen.

**Supplementary Figure 6. High throughput antibody escape selection does not detect escape of RVFV strain MP-12 from mAb RVFV-140, RVFV-268 or the combination of the two mAbs during a single-round infection.** RVFV strain MP-12 was premixed with human antibodies **A)** RVFV-140, **B)** RVFV-268 or **C)** the combination of the two mAbs before adding the mixture to a Vero cell monolayer in a 96-well plate format. Each well represents a tested condition to maximize detection of escape. In the bottom right-hand corner, cells-only and [cells + virus]-only controls were included.

**Supplementary Figure 7. Gating strategy used for flow cytometric analysis of mutations in naturally occurring variants of RVFV.** A) Representative plot of all events captured and first gate on events that represent cells using SSC-H vs FSC-H. B) Representative plot for live cells showing # Events vs VL1-H channel when strained with Violet Live/Dead stain before being treated with fixative and stained with monoclonal antibodies and secondary. C) Representative plots showing live cells that were either transiently transfected with Gn encoding plasmids of variants and untransfected control cells strained with Gn specific monoclonal antibody and PE-conjugated secondary. Parameters for positivity was cut off for events above 10^5 BL2-H channel for the detection of the PE-conjugated goat anti-human IgG secondary.

**Supplementary Figure 8. Monotherapy is effective at mid-range doses against WT infection in the BALB/c mouse model. A)** A single dose of mAb was administered by the IP route to mice (n = 8 per group) on 2 d.p.i. Doses administered for RVFV-140: 200 µg, 60 µg, or 20 µg. Doses administered for RVFV-268: 200 µg, 20 µg, 2 µg, or 0.2 µg. Virus challenge was by SC inoculation of 300 PFU of RVFV strain ZH501. RVFV-268, RVFV-140, or DENV-2D22 (isotype-matched negative control mAb) were tested in a sequential dose-down study design in this stringent therapeutic model of infection. Kaplan-Meier survival plots were analysed statistically using a log-rank (Mantel-Cox) test where treated animals (****P*<0.001, ***P*<0.01, **P*<0.05) were compared to animals treated with the DENV-2D22 negative control. Weight graphs reflect the group means and standard error of the means of the percent change in weight of animals relative to the weight obtained the day of virus challenge. Sham-infected no virus controls are shown. Median survival for each condition tested: normal control – Undefined; DENV-2D22 – 5.5 days; 200 µg RVFV-140 – Undefined; 60 µg RVFV-140 – Undefined days; 20 µg RVFV-140 – Undefined; 200 µg RVFV-268 – Undefined; 20 µg RVFV-268 – Undefined; 2 µg RVFV-268 – Undefined; 0.2 µg RVFV-268 – Undefined. P values for each condition tested compared to the DENV-2D22 control treated group using a Log-rank (Mantel-Cox) test: no-virus control – 0.0100; 200 µg RVFV-140 – 0.0019; 60 µg RVFV-140 – 0.0004; 20 µg RVFV-140 – 0.0012; 200 µg RVFV-268 – 0.0016; 20 µg RVFV-268 – 0.0004; 2 µg RVFV-268 –0.0016; 0.2 µg RVFV-268 –0.0117. **B**) Table indicating single nucleotide polymorphisms detected and their respective frequencies that have the capacity to induce an amino acid (AA) shift in the indicated positions from the brain and liver derived virus from a mouse who died of late-stage neurological complications despite being treated with RVFV-140. Input wild type (WT) virus sequence is from Genbank ID: DQ380200, and amino acid positioning begins at the first coding sequence of the M segment.

**Supplementary Figure 9. RVFV-specific mAbs are effective in reducing viral titers in various organs at low doses.** Viral titer data were obtained using an infectious cell culture assay in technical triplicate to assess efficacy of mAbs in the therapeutic setting. Viral cytopathic effect was used to calculate 50% endpoints. Lower limits of detection (LOD) were 1.49 log_10_ 50% cell culture infectious dose (CCID_50_)/mL for serum or 2.1 log_10_ CCID_50_/g tissue. In samples presenting with virus below the limit of detection (LOD), the representative value of LOD was assigned for analysis. Human mAb DENV 2D22 (specific to an unrelated target, dengue virus) was used as the negative control mAb. Four animals per group were sacrificed on 3 d.p.i. for analysis of virus in serum, liver, and spleen. The dotted line represents the LOD. Data were analysed using an ordinary one-way ANOVA correcting for multiple comparisons using a Dunnett’s post-test to compare the differences in viral titer (***P*<0.01, **P*<0.05). Each uniquely colored shape represents a single animal.

**Supplementary Figure 10. Antibody combination is effective in reducing viral titers in various organs in the therapeutic setting.** Assessment of viral titers in harvested organs in the dose-down study were performed as in the monotherapy study above. Tested combinations of mAbs were given to BALB/c mice at 2 d.p.i. to test the therapeutic efficacy, and PBS was used as the negative control in this experiment. Four animals per group were sacrificed on 3 d.p.i. for analysis of serum, liver, or spleen. The dotted line represents the LOD. Data were analysed using an ordinary one-way ANOVA correcting for multiple comparisons using a Dunnett’s post-test to compare the differences in viral titer (***P*<0.01, **P*<0.05). Each uniquely colored shape represents a single animal.

**Supplementary Table 1.** Crystallographic data collection and refinement statistics.

| **Data Collection Statistics** | **RVFV Gn−Fab RVFV-268** | **Fab RVFV-268** |
| --- | --- | --- |
| Beamline | DLS I04 | DLS I04 |
| Wavelength (Å) | 0.9795 Å | 0.9795 Å |
| Space Group | *P2_1_ 2_1_ 2_1_* | *P2_1_ 2_1_ 2_1_* |
| Cell dimensions  *a, b, c* (Å) | 73.1, 98.6, 123.1 | 54.4, 70.4, 129.9 |
| 𝛼, β, 𝛾 (°) | 90, 90, 90 | 90, 90, 90 |
| Resolution range (Å) | 61.57**−**3.50 | 47.74**−**1.62 |
| Rmerge | 0.56 (>1.0) | 0.10 (>1.0) |
| I/σ (I)* | 2.7 (0.5) | 11.4 (0.4) |
| CC_1/2*_ | 0.992 (0.789) | 1.0 (0.400) |
| Completeness (%)* | 100 (100) | 99.1 (94.1) |
| Multiplicity* | 13.0 (13.3) | 13.3 (10.9) |
| **Refinement Statistics** |  |  |
| Resolution (Å) | 61.57**−**3.50 | 41.74**−**1.62 |
| No. reflections | 10,512 | 63,726 |
| *R_work_/R_free_* | 0.247/0.279 | 0.202/0.219 |
| **No. atoms**  Protein  Ligand  Water | 5,547  0  0 | 3,214  18  201 |
|  |  |  |
| **Average B-factors**  Protein  Ligand  Water | 80.8  n/a  n/a | 33.0  83.5**  35.6 |
|  |  |  |
| **Ramachandran (%)**  Favored  Allowed  Outlier | 96.2  3.8  0 | 98.6  1.4  0 |
|  |  |  |
| **Root mean square deviations (RMSD)**  Bond lengths (Å)  Bond angles (°) | 0.004  0.82 | 0.011  1.11 |
|  |  |  |

*The value for the highest-resolution shell is shown in parentheses.

** Citric acid derived from the precipitant was observed in the crystal.
