# Supplementary material for: Multifunctional human monoclonal antibody combination mediates protection against Rift Valley fever virus at low doses
Source: Nat Commun. 2023 Sep 13;14:5650. doi: 10.1038/s41467-023-41171-3 (PMC10499838; doi:10.1038/s41467-023-41171-3)
Supplement: Supplementary file 2 — Reporting Summary [file 41467_2023_41171_MOESM2_ESM.pdf]

Corresponding author(s): James E. Crowe, Jr.

Last updated by author(s): August 2, 2023

Reporting Summary

Nature Portfolio wishes to improve the reproducibility of the work that we publish. This form provides structure for consistency and transparency in reporting. For further information on Nature Portfolio policies, see our Editorial Policies and the Editorial Policy Checklist.

Statistics

For all statistical analyses, confirm that the following items are present in the figure legend, table legend, main text, or Methods section.

n/a	Confirmed	
☐	☒	The exact sample size (*n*) for each experimental group/condition, given as a discrete number and unit of measurement
☐	☒	A statement on whether measurements were taken from distinct samples or whether the same sample was measured repeatedly
☐	☒	The statistical test(s) used AND whether they are one- or two-sided<br>*Only common tests should be described solely by name; describe more complex techniques in the Methods section.*
☒	☐	A description of all covariates tested
☐	☒	A description of any assumptions or corrections, such as tests of normality and adjustment for multiple comparisons
☐	☒	A full description of the statistical parameters including central tendency (e.g. means) or other basic estimates (e.g. regression coefficient) AND variation (e.g. standard deviation) or associated estimates of uncertainty (e.g. confidence intervals)
☐	☒	For null hypothesis testing, the test statistic (e.g. *F*, *t*, *r*) with confidence intervals, effect sizes, degrees of freedom and *P* value noted<br>*Give P values as exact values whenever suitable.*
☒	☐	For Bayesian analysis, information on the choice of priors and Markov chain Monte Carlo settings
☒	☐	For hierarchical and complex designs, identification of the appropriate level for tests and full reporting of outcomes
☒	☐	Estimates of effect sizes (e.g. Cohen's *d*, Pearson's *r*), indicating how they were calculated

Our web collection on statistics for biologists contains articles on many of the points above.

Software and code

Policy information about availability of computer code

Data collection

X-ray diffraction data collection; Diamond Light Source Generic Data Acquisition (GDA) software (9.21) on beamline i04 with a Dectris Eiger2XE 16M detector. MP-12 Virus-infected cells BioSpot™ ImmunoCapture Analyzer 7.0.18.1 (https://immunospot.com/biospot-software.html). Bio-layer interferometry; Pall FortéBio Octet Red96.

Negative-stain electron microscopy imagine was recorded on a Gatan US4000 4kx4k CCD camera using a FEI TF20 (TFS) transmission electron microscope operated at 200 keV and control with SerialEM. All images were taken at 50,000X magnification with a pixel size of 2.18 angstroms/pixel in low-dose at a defocus of 1.5 to 1.8 mm.

Data analysis

X-ray data scaling and reduction; xia2 (v3.8.0-g3d57088-dials-3.8), X-ray data phasing and molecular replacement on PHASER, Protein structure model building; Coot (v0.9.4.1), PHENIX (v1.19.2_4158), Protein structure visualization; ChimeraX (v1.2.5), quality assessment on MolProbity. Data representation and statistical analysis; GraphPad Prism software v9 (GraphPad Prism; RRID: SCR_002798). Flow cytometry data; iQue Forecyt® Software v9.0 (https://www.sartorius.com/en/products/flow-cytometry/flow-cytometry-software). MP-12 escape selection and neutralization; RTCA software version 2.1.0 (Agilent/Acea Biosciences; RRID: SCR_014821). MP-12 Virus-infected cells BioSpot™ BioSpot 7.0.18.1 professional Software Suite. Affinity data; Analysis HT (12.2.0.2). Synergy analysis; SynergyFinder software (R-3.8.2). Sequence analysis of viral sequencing; Geneious Prime V2020.1.2 (https://www.geneious.com/).

Negative stain electron microscopy image acquisition and processing was performed using the cryoSPARC software package version 3.3.1. The images were imported, CTF-estimated and particles were picked automatically. The particles were extracted with a box size of 200 pix and binned to 100 pix (4.36 Å/pixel) and multiple rounds of 2D class averages were performed to achieve clean datasets. The final dataset was used to generate an initial 3D volume and the volume was refined for the final map at the resolution of ~18 Å. Model docking to the EM map

was done in Chimera. UCSF ChimeraX v1.3 was used to visualize molecular structures and freely available from https://www.cgl.ucsf.edu/chimerax/.

For manuscripts utilizing custom algorithms or software that are central to the research but not yet described in published literature, software must be made available to editors and reviewers. We strongly encourage code deposition in a community repository (e.g. GitHub). See the Nature Portfolio guidelines for submitting code & software for further information.

Data

Policy information about availability of data

All manuscripts must include a data availability statement. This statement should provide the following information, where applicable:

- Accession codes, unique identifiers, or web links for publicly available datasets
- A description of any restrictions on data availability
- For clinical datasets or third party data, please ensure that the statement adheres to our policy

Atomic coordinates and structure factors of the RVFV Gn + Fab RVFV-268 complex and RVFV-268 Fab Apo have been deposited in the PDB (accession code PDB ID 8AWM and 8AWL Materials used in this study will be made available but may require execution of a Materials Transfer Agreement. All relevant and source data for each main text figure are available with this manuscript. Supplemental data are available upon request. The following sequences were used to design constructs for protein expression: WT Gn protein was based on the sequence corresponding to GenBank accession number JQ068143.1 [https://www.uniprot.org/uniprotkb/H9BSP3/entry] and UniProt accession no. P21401 [https://www.uniprot.org/uniprotkb/P21401/entry]. The following sequence was used for viral isolate sequence comparison: Genbank ID: DQ380200 [https://www.ncbi.nlm.nih.gov/nuccore/DQ380200]. The atomic models used for data visualization were RVFV Gn (PDB: 5Y0W) [https://www.rcsb.org/structure/5Y0W] and of a human Fab (PDB: 5ZMJ) [https://www.rcsb.org/structure/5ZMJ]. The atomic structures for comparative representation used in Supplemental Figure S3 were: PDB: 6IEK [https://www.rcsb.org/structure/6IEK], PDB: 6IEA [https://www.rcsb.org/structure/6IEA], PDB: 6IEB [https://www.rcsb.org/structure/6IEB], PDB: 6IEC [https://www.rcsb.org/structure/6IEC] from Wang et al., 2019, and PDB: 6I9I [https://www.rcsb.org/structure/6I9I] from Allen, et al., 2018. Modeling of RVFV-268 onto higher order pentameric Gn-GC was with PDB: 6F9F [https://www.rcsb.org/structure/6f9f], and modeling with the icosahedral assembly of RVFV Gn-Gc was with PDB: 6F9B [https://www.rcsb.org/structure/6F9B]. To aid in the strucutre of RVFV Gn -Fab-268 complex, unliganded RVFV Gn was used PDB: 6F8P [https://www.rcsb.org/structure/6F8P].

Human research participants

Policy information about studies involving human research participants and Sex and Gender in Research.

Reporting on sex and gender	N/A
Population characteristics	N/A
Recruitment	N/A
Ethics oversight	N/A

Note that full information on the approval of the study protocol must also be provided in the manuscript.

Field-specific reporting

Please select the one below that is the best fit for your research. If you are not sure, read the appropriate sections before making your selection.

☒ Life sciences ☐ Behavioural & social sciences ☐ Ecological, evolutionary & environmental sciences

For a reference copy of the document with all sections, see nature.com/documents/nr-reporting-summary-flat.pdf

Life sciences study design

All studies must disclose on these points even when the disclosure is negative.

Sample size	For animal studies, sample-size calculations for primary survival endpoint were performed to power each study to have a type I error of 0.05 (95% confidence) and 80% power with a 50% or greater expected survival outcome in protected groups and an expected survival of 0% in placebo/negative control groups. Key experiments that included in vitro measurements of antibody binding, and virus neutralizing activities were carried out with two or more independent study replicates.
Data exclusions	No data were excluded from analysis.
Replication	Repeated studies are noted in figure legends. All key results in the overall study were repeated. No studies have been reported that failed upon repetition. Negative control antibody for the animal study was DENV-2D22, and for binding and neutralization assays was CCHF-245 IgG1, for no specific reason other than choosing an antibody that neutralizes more closely related virus (CCHFV). These antibodies account for nonspecific binding, neutralization, and isotype performance in animals. Results for the human RVFV specific antibodies were consistent with what was reported in a previous publication (https://www.pnas.org/doi/10.1073/pnas.2025642118), except for RVFV-429 which was previously assessed as a hybridoma derived antibody, but in this manuscript, the recombinant form of this antibody's neutralization was assessed. These studies indicate a high level of reproducibility.

Randomization	The principle of randomization is not relevant as this is an observational study.
Blinding	The investigators were not blinded for these studies.

Reporting for specific materials, systems and methods

We require information from authors about some types of materials, experimental systems and methods used in many studies. Here, indicate whether each material, system or method listed is relevant to your study. If you are not sure if a list item applies to your research, read the appropriate section before selecting a response.

Materials & experimental systems

n/a	Involved in the study
☐	☒ Antibodies
☐	☒ Eukaryotic cell lines
☒	☐ Palaeontology and archaeology
☐	☒ Animals and other organisms
☒	☐ Clinical data
☒	☐ Dual use research of concern

Methods

n/a	Involved in the study
☒	☐ ChIP-seq
☐	☒ Flow cytometry
☒	☐ MRI-based neuroimaging

Antibodies

Antibodies used	For MP-12 neutralization assays: 1:3,000 dilution of anti-mouse IgG HRP-conjugated secondary antibodies (Promega, W402B LOT:0000262777). For flow cytometric assays, a PE-conjugated goat anti-human-IgG (Southern Biotech, 2040-09, 1:500 dilution) was used to label bound antibody. Previously discovered RVFV glycoprotein-specific monoclonal antibodies were described in https://www.pnas.org/doi/10.1073/pnas.2025642118. CCHF-245 is an unpublished CCHF specific neutralizing antibody. DENV-2D22 is a previously published antibody generated in the Crowe lab.
Validation	Previously discovered RVFV specific monoclonal antibodies were validated via antigen binding, virus neutralization, and in a previous paper in vivo protection studies (https://www.pnas.org/doi/10.1073/pnas.2025642118). CCHF-245 is an unpublished antibody against CCHFV was validated for binding and neutralization on vecVLPs. DENV-2D22 was validated in the lab. All other antibodies are commercially available. Antibodies used in a specific species or application have been appropriately validated by manufacturers and this information is provided on their website and information datasheets as follows:<br>Goat anti-mouse IgG-HRP (https://www.promega.com/products/protein-detection/primary-and-secondary-antibodies/anti_mouse-igg-h-and-l-hrp-conjugate/);<br>PE-conjugated goat anti-human-IgG (https://resources.southernbiotech.com/techbul/2040.pdf)

Eukaryotic cell lines

Policy information about cell lines and Sex and Gender in Research

Cell line source(s)	In this study we used the following cell lines: Vero (ATCC Cat# CCL-81), Vero E6 (ATCC Cat# CLR-1586), Expi293F (ThermoFisher Scientific, A1452), Sf9 cells in Sf-900 II SFM (Thermo Fisher Scientific, 11496015) (used to generate Gn recombinant protein that was reported in a previous study Chapman, et al 2019),, SH-SY5Y (ATCC: The Global Bioresource Center, CRL-2266), Hep G2 (HEPG2) (ATCC: The Global Bioresource Center, HB-8065), and HEK-293 (ATCC: The Global Bioresource Center, CRL-3216)
Authentication	None of the cell lines used were authenticated.
Mycoplasma contamination	Expi293F, ExpiCHO, and Vero cell lines were tested and confirmed negative for mycoplasma contamination. HepG2, HEK293, and SH-SY5Y from ATCC were only passaged three times and not assessed for mycoplasma. Sf9 cells were not assessed for mycoplasma contamination.
Commonly misidentified lines<br>(See ICLAC register)	None

Animals and other research organisms

Policy information about studies involving animals; ARRIVE guidelines recommended for reporting animal research, and Sex and Gender in Research

Laboratory animals	Seven- to eight-week-old male and female BALB/c mice were obtained from Charles River Laboratories (Wilmington, MA). Mice were housed in microisolator cages and provided water and food ad libitum. Mice were housed in the biosafety level-3 enhanced laboratory in a GM500 Green Line IVC system (Tecniplast SpA, Italy) in individually ventilated cages and fed Harlan Lab Block and tap water ad libitum. Room air temperature was 72 +/- 4 degrees Fahrenheit with 30-70% air humidity and the dark/light cycle was 12 hours/12 hours.

Wild animals	This study did not involve wild animals.
Reporting on sex	These studies used groups that had 4 male and 4 female for the survival assessment (total n=8) and for titers for viremia 2 male and 2 female (total n=4), and for sham n =5 with 2 sacrificed for viral titer controls. Methods for assigning sex in the study was 50:50, half male and half female. We did not perform sex-based analysis given that the groups were typically uniformly lethal and animals died within a typical time frame.
Field-collected samples	The study did not involve samples collected from the field.
Ethics oversight	The mouse RVFV challenge efficacy studies were approved by the Utah State University Institutional Biosafety Committee and conducted in Select Agent-approved animal (A)BSL-3+ facilities

Note that full information on the approval of the study protocol must also be provided in the manuscript.

Flow Cytometry

Plots

Confirm that:

☒ The axis labels state the marker and fluorochrome used (e.g. CD4-FITC).

☒ The axis scales are clearly visible. Include numbers along axes only for bottom left plot of group (a 'group' is an analysis of identical markers).

☐ All plots are contour plots with outliers or pseudocolor plots.

☒ A numerical value for number of cells or percentage (with statistics) is provided.

Methodology

Sample preparation	Briefly, Expi293F cells transiently expressing RVFV Gn (expressed for 48 hours) were washed and stained with Live/Dead fixable violet dead cell stain (Thermo Fisher Scientific; L34955) per manufacturer's protocol. Cells were washed and fixed using Cytofix/Cytoperm (Becton Dickinson and Co; 554714). Cells were diluted to 50,000 cells per 30 µL in a 96 V-bottom plate. Cells were incubated with human antibodies at 20 µg/mL in perm/wash buffer for 30 min at room temperature. Cells were washed and stained with Cells were washed with 100 µL of perm/wash buffer, spun down, and then stained with goat anti-human IgG-PE (SouthernBiotech; 2040-09) at a 1:500 dilution in perm/wash buffer for 30 min at room temperature. Cells were then washed with 100 µL of perm/wash buffer and spun down and resuspended in 30 µL of FACS buffer. Staining was analyzed using an iQue flow cytometer (Intellicyt). Background values were determined from binding of labeled secondary mAb to untransfected Expi293F cells. Cells were gated for viability and the positive signal was gated using a similarly prepared human mAb to an unrelated antigen (clone CCHF- 245) as a negative control. Results were expressed as the percent of positive cells over total viable cell counts.
Instrument	iQue Screener PLUS
Software	iQue Forecyt® Software
Cell population abundance	For a typical sample, approximately 40-70% of each sample fell into the "PE+" gate, compared with <1% of the untransfected Expi293F population stained with the same concentration of antibody.
Gating strategy	Cells were first gated by forward and side scatter and dead cells were excluded using a viability dye (violet Live/Dead). The gate for the glycoprotein-specific subset (PE+) was placed based on staining of untransfected Expi293F cells. The gating strategy is shown in the supplemental figures.

☒ Tick this box to confirm that a figure exemplifying the gating strategy is provided in the Supplementary Information.
